# Canine colorectal proliferative lesions: a retrospective study of 217 cases

**DOI:** 10.1186/s12917-025-04567-5

**Published:** 2025-03-05

**Authors:** Joanna Fiedorowicz, Katarzyna Paździor-Czapula, Iwona Otrocka-Domagała

**Affiliations:** Department of Pathological Anatomy, Faculty of Veterinary Medicine, University of Warmia and Mazury in Olsztyn, Oczapowskiego 13, 10-719 Olsztyn, Poland

**Keywords:** Dog, Large intestine, Rectum, Colorectal cancer, Adenocarcinoma

## Abstract

**Background:**

Colorectal proliferative lesions are not common in dogs. However, recently we have observed an increase in the number of diagnosed cases and a lack of publications providing current epidemiological data on lesions of the large intestine in dogs. The aim of this study was a retrospective analysis of 217 canine colorectal non-neoplastic and neoplastic nodular lesions, and assessment of the frequency of occurrence of individual lesions and whether there is a risk of their occurrence depending on age, sex, or dog breed.

**Results:**

Half of the cases (52.5%) were malignant tumours with a significant predominance of adenocarcinoma (42.9%). In the group of malignant non-epithelial lesions, lymphoma and sarcomas predominated (4.1% and 4.1%, respectively) followed by three cases of plasmacytoma. Benign neoplastic tumours constituted almost one-third of all cases (26.7%) with obvious dominance of adenoma (24.0%). Benign mesenchymal tumours were represented only by leiomyoma (2.8%). The non-neoplastic lesions were represented by a heterogeneous group of polyps (20.3%) with a slight advantage of hyperplastic type (9.7%) and less numerous inflammatory, fibroblastic, lymphoid, and hamartomatous polyps. One case of ganglioneuromatosis in hamartomatous polyp was diagnosed. The vast majority of lesions, both non-neoplastic and neoplastic, were found in the rectum. French Bulldogs were the most numerous breeds in our study, especially among adenomas. Furthermore, benign tumours were diagnosed in younger animals than malignant tumours.

**Conclusions:**

The results of our research provided new data expanding knowledge about the epidemiology of colorectal neoplastic and non-neoplastic proliferative lesions in dogs. Our results indicate that the majority of colorectal proliferative lesions in dogs are malignant, which is alarming. French Bulldogs could possibly be predisposed to proliferative lesions of the large intestine, and this predisposition was statistically confirmed in adenomas. Moreover, benign tumours may occur in animals as young as 1–2 years old.

## Background

Over the last several decades, an increase in the frequency of diagnosed colorectal neoplasms and non-neoplastic polyps has been observed in humans [28]. It has been proven that this increase is related to dietary habits, an inactive lifestyle, processed food and environmental pollution. This high mortality rate of colorectal cancer causes the interest of a lot of scientists and encourages them to find the best option to prevent development of this tumour. One of the methods which is widely used in human medicine for screening tests is routine colonoscopy, which is offered in developed countries to high-risk groups. This examination is able to detect asymptomatic lesions from the large intestine and has a positive effect on diagnosis at an early stage of cancers [26, 43]. Companion animals, especially dogs, living in the same environment as man are prone to the same civilization diseases, which is reflected in the increase in the number of tumoral colorectal diseases [23]. In addition to environmental risk factors, genetic and hereditary predispositions, as well as age, sex and breed are also under consideration in the development of intestinal proliferative lesions in dogs [32, 57]. Breed related development of colorectal inflammatory polyps with risk of progression to adenoma and adenocarcinoma has been shown in middle-aged Miniature Dachshunds in Japan [48, 54]. In Jack Russel Terriers hereditary gastrointestinal polyposis was confirmed with gastric and colorectal distribution. While hyperplastic polyps, adenomas (tubular, tubulopapillary, papillary) and adenocarcinomas (tubular, tubulopapillary, papillary) were observed in the stomach in this breed, only papillary adenocarcinomas were observed in the colon and rectum [61].

The canine large intestine is divided into three main parts: cecum, colon, and rectum, similar to humans and all mammals. The term 'colorectal' is widely used for this region, as the border between the rectum and colon is often indistinct, both macroscopically and in histopathological classification. However, based on certain studies in human medicine, notable differences exist between colon cancer and rectal cancer in terms of prognosis, metastasis, and biology, with worse outcomes associated with rectal cancer. [30, 40]. Interestingly, the opposite situation is suspected in dogs, where the vast majority of large intestine adenocarcinomas are found more commonly in the rectum than in the colon, and it has been suggested that they have a better prognosis [7, 35]. Unfortunately, the number of studies is insufficient to confirm this hypothesis, and more research needs to be conducted.

According to the current classification of tumours in domestic animals, canine intestinal neoplasms are divided into tumours of epithelial and mesenchymal origin. Epithelial tumours include adenomas, adenocarcinomas and neuroendocrine carcinomas, while mesenchymal tumours include lymphomas, plasmacytomas, mast cell tumours, non-angiogenic, non-lymphogenic intestinal mesenchymal tumours (NIMTs), gastrointestinal stromal tumours (GISTs), leiomyomas, leiomyosarcomas, intestinal neurogenic tumours, fibrosarcomas, myxosarcomas, extraskeletal osteosarcomas and angiogenic tumours. Non-neoplastic proliferative lesions in dogs most frequently develop in the rectum as hyperplastic polyps, caused by abnormal mucosal maturation, inflammatory polyps, caused by chronic inflammation, and multiple polypoid hamartomas [34, 36].

The vast majority of colorectal malignant neoplasms in dogs are adenocarcinomas, followed by lymphomas, leiomyosarcomas, haemagiosarcomas and plasmacytomas [23, 49, 53]. The most frequently diagnosed benign tumours in this location are adenomas (adenomatous polyps), leiomyomas and fibromas [1, 23, 49]. Both malignant and benign neoplasms, especially of epithelial origin, may grow as single or multiple polypoid lesions of various diameters, variably involving the mucosa, and in the case of malignancies, also the submucosa and muscularis of the intestine. Therefore, on the basis of endoscopic examination, these lesions are generally referred to as colorectal polyps. Meanwhile, histopathologically, intestinal polyps are defined as non-neoplastic sessile or pedunculated exophytic growths [54]. According to the current histopathological classification, non-neoplastic colorectal polyps in humans are classified as: hyperplastic (metaplastic), hamartomatous (Peutz-Jeghers-type), juvenile, inflammatory and lymphoid ([19], [9]), and the similar classification is used in animals. In humans, the most common are hyperplastic polyps, which have a low malignant potential [51]. Juvenile, inflammatory, and lymphoid polyps have a low risk of neoplastic transformation, while in the case of Peutz-Jeghers-type polyps, the probability of malignant transformation is high [6, 13, 25]. In dogs, hyperplastic and inflammatory polyps are the most common. Numerous studies have shown that, as in humans, the growth of inflammatory polyps in dogs is stimulated by chronic inflammatory bowel disease, which is diagnosed with the increased frequency in animals [39].

The aim of this retrospective study was to analyze the incidence of neoplastic and non-neoplastic colorectal proliferative lesions in dogs, depending on age, sex and breed. The results of our research will provide new data expanding knowledge about the epidemiology of colorectal neoplasms and non-neoplastic polyps in dogs and may also help to determine the possible risk factors for their development.

## Material and Methods

This study included 217 samples of canine colorectal proliferative lesions sent for histopathological examination during 2015–2022. All tissue samples were obtained during surgery or colonoscopy, fixed in 10% buffered formalin, processed routinely, embedded in paraffin, cut into 3 µm sections and stained with Mayer's haematoxylin and eosin (HE). 

When the diagnosis was not obtained on routine HE staining, additional immunohistochemistry was applied for definitive diagnosis (Table 1). Immunohistochemical examination was performed manually and preceded by heat-induced antigen retrieval in Tris–EDTA buffer (pH 9.0) using PT-Link (Dako, Glostrup, Denmark). Primary antibodies included: CD3 (polyclonal rabbit anti-human, dilution 1:100, Dako), CD20 (monoclonal rabbit anti-human, clone SP32, dilution 1:100, Abcam, Cambridge, UK), CD79a (monoclonal mouse anti-human, clone HM57, dilution 1:100, Bio-Rad Laboratories Inc., Hercules, CA), vimentin (monoclonal mouse anti-porcine, clone V9, dilution 1:100, Dako), desmin (monoclonal mouse anti-human, clone D33, dilution 1:50, Dako), c-Kit (CD117; polyclonal rabbit anti-human, dilution 1:400, Dako), α-smooth-muscle-actin (α-SMA; monoclonal mouse anti-human, clone 1A4, dilution 1:500, Dako), neuron-specific enolase (NSE; monoclonal mouse anti-human, clone BBS/NC/Vi-H14, dilution 1:100, Dako), MUM-1 (monoclonal mouse anti-human, clone MUM1p, dilution 1:50, Dako), HLA-DR α-chain (MHC II; monoclonal mouse anti-human, clone TAL.1B5, dilution 1:20, Dako), S-100 (polyclonal rabbit, anti-bovine, dilution 1:50, Dako), pancytokeratin (monoclonal mouse anti-human, clone AE1/AE3/PCK26, ready-to-use, Ventana, Tucson, AZ), mast cell tryptase (monoclonal mouse anti-human, clone AA1, dilution 1:200, Dako) DOG1 (monoclonal rabbit recombinant, Anti-TMEM16A, clone SP31, dilution 1:100, Abcam). Visualization system was based on the immunoperoxidase method (ImmPRESS HRP Horse Anti-Rabbit IgG Polymer Reagent – for primary rabbit antibodies, Vector, Newark, CA and ImmPRESS HRP Horse Anti-Mouse IgG Polymer Reagent for primary mouse antibodies, Vector) with 3.3-diaminobenzidine (DAB) as the substrate (ImmPACT DAB, SK-4105, Vector). The slides were counterstained with Mayer’s hematoxylin. Positive and negative control slides were processed together with the evaluated sections. Each case was evaluated and classified by three veterinary pathologists (IOD, KPC, JF) based on the World Health Organization (WHO) International Histologic Classification of Tumors of Domestic Animals [21] and according to classification include at Munday et.al 2007.

**Table 1:** Antibody panels used in immunohistochemistry for selected tumours

Tumour	Number of cases	Antibodies
B-cell lymphoma	6	CD3-, CD20 + , CD79a +
T-cell lymphoma	3	CD3 + , CD20-, CD79a-
Fibrosarcoma	4	vimentin + , desmin-, S-100 -, c-Kit-, α-SMA-
Leiomyosarcoma	2	vimentin + , α-SMA + , desmin -, S-100-, c-Kit-, DOG1 -
Gastrointestinal stromal tumour (GIST)	2	DOG1 + , c-Kit + , vimentin + , α-SMA + , desmin-, S-100-
Non-Gist, non-smooth muscle NIMT (NIMTs)	1	vimentin + , S100 + , NSE + , CD3-, CD20-, CD79a-, MHC II-, MUM1-, desmin-, c-Kit-, pancytokeratin-, α-SMA-

### Statistical analysis

To assess the influence of sex, breed, and age on the occurrence of proliferative lesions in the canine large intestine, groups were compared as non-neoplastic lesions vs. neoplastic lesions, and benign tumours vs. malignant tumours. Statistical analysis was carried out using a Fisher’s exact test or the chi-square test for nominal data and Student’s T test for continuous data. Verification of normality of distribution for age was based on Shapiro–Wilk test and analysis of skewness and kurtosis values. To identify predictors for the occurrence of neoplastic lesions (non-neoplastic vs neoplastic) and (benign tumours vs malignant tumours) additionally univariate logistic regression was performed. All data were analyzed using R statistical software (version 4.1.2; R Core Team, 2022; R Foundation for Statistical Computing, Vienna, Austria) and results were considered significant when *P*-value was less than 0.05 (*p* < 0.05).

## Results

Among collected cases of proliferative colorectal lesions in dogs, 123 cases were diagnosed in males (56.7%) and 94 cases in females (43.3%).

The rectum was the typical location of all lesions diagnosed in this study, constituting 97.6% (41/42) of non-neoplastic lesions, 90.9% (50/55) of benign tumours, and 92.3% (96/104) of malignant tumours. Whereas, the colon was much less frequently affected, with 2 cases of benign tumours (3.6%, 2/55) and 7 cases of malignant tumours (6.8%, 7/103). The colorectal junction was identified by clinicians in 3 cases of adenomas and 1 case of non-neoplastic lesion and adenocarcinoma. Unfortunately, based on information from clinicians and histopathological review, in several cases we could not specify the localization of the large intestine: 3 cases of non-neoplastic lesions, 3 cases of benign tumours, and 10 cases of malignant tumours.

**Fig.1:**
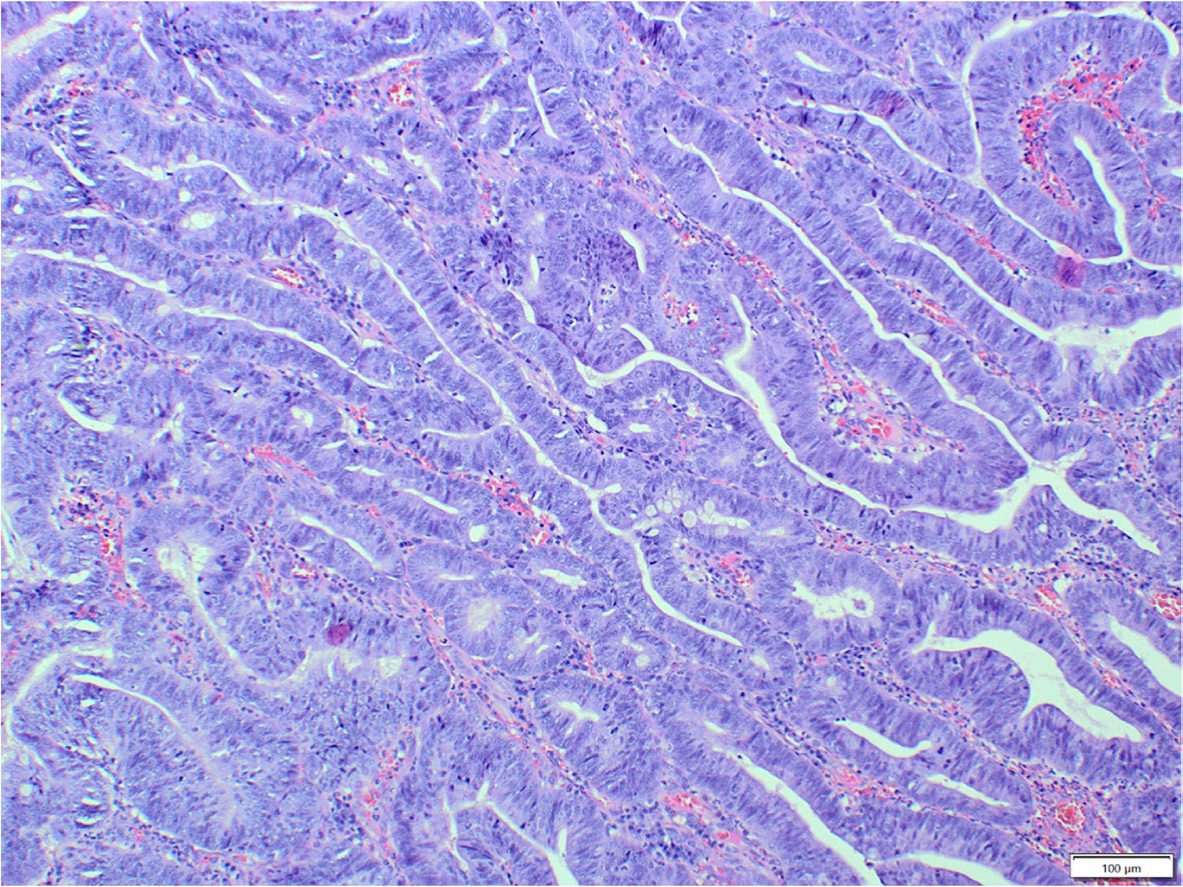
Dog, adenocarcinoma, large intestine. The tumour cells are cylindrical, polygonal with moderate to high anisocytosis and anisokaryosis and many mitoses. Loss of proper architecture of glands with multilayer formation. Reduced stroma with congestion, infiltrated by lymphocytes and plasma cells. Hematoxylin and eosin (HE)

The majority of proliferative lesions constituted malignant tumours (52.5%, 114/217), with the most frequent adenocarcinomas (42.9%, 93/217). Adenocarcinomas were further subtyped into papillary (72 cases, Fig. 1), mixed (12 cases), tubular (7 cases), mucinous (one case) and adenosquamous carcinoma (one case). Adenocarcinomas occurred with a slightly higher incidence in males (55/93) than in females (38/93), but the difference was not statistically significant. The age ranged from 2 to 16 years (mean age: 8 years, standard deviation SD ± 3.22). The most common breeds diagnosed with adenocarcinoma were French Bulldogs (10/67), Yorkshire Terriers (7/67) and crossbreeds (7/67). Unfortunately, these data were incomplete in several cases: 7 regarding the age and 26 regarding the breed.

**Fig.2:**
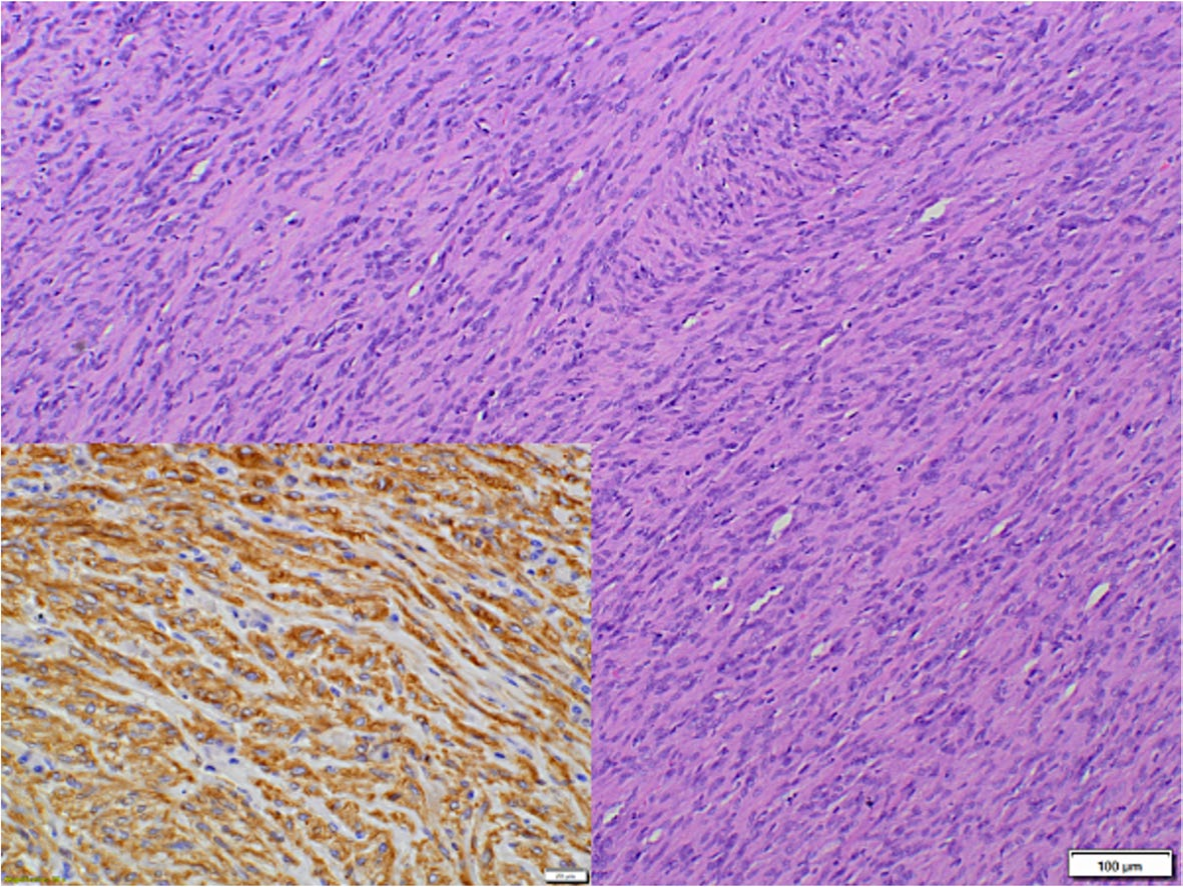
Dog, Gastrointestinal stromal tumour, large intestine. Tumour cells are spindle-shaped and densely packed with moderate to high anisocytosis and anisokaryosis, forming palisades and herringbone pattern. Hematoxylin and eosin (HE). Tumour cells show cytoplasmic expression for DOG1 (inset)

**Fig. 3:**
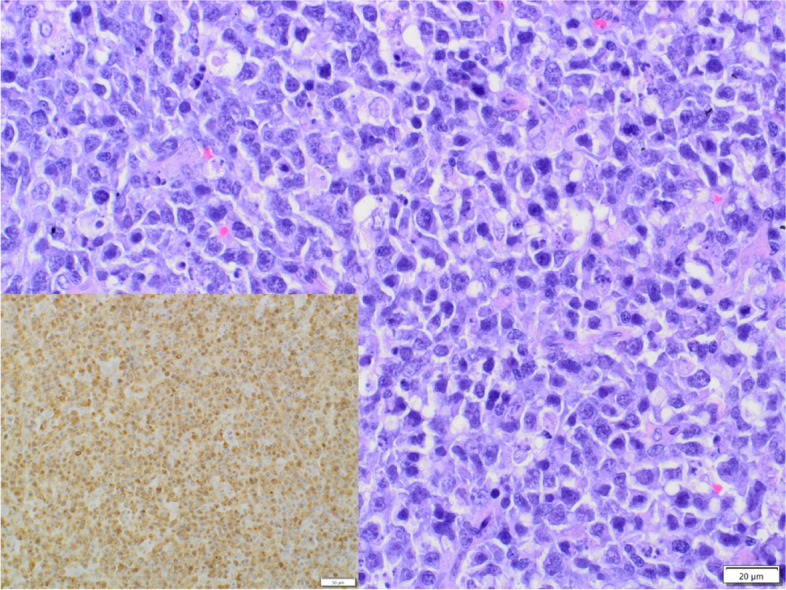
Dog, large B-cell lymphoma, large intestine. Neoplastic lymphocytes show high mitotic activity. Hematoxylin and eosin (HE). Tumour cells show cytoplasmic expression for Cd79a (inset)

Malignant tumours of non-epithelial origin were far less common than adenocarcinomas, and constituted 9.7% (21/217) of cases, including 9 cases of sarcomas, 9 cases of lymphomas and 3 cases of plasmacytomas. In all sarcomas and lymphomas, the final diagnosis was supported by immunohistochemistry. Sarcomas were represented by 4 cases of fibrosarcoma, 2 cases of leiomyosarcoma, 2 cases of GIST (Fig. 2) and one case of non-GIST, non-smooth muscle NIMT. In the group of sarcomas, sex distribution was almost equal, with an age ranged from 6 to 16 years (mean age: 10.9y.; SD ± 3.4). Fibrosarcomas were confirmed in 3 males and one female, with a mean age of 10.7 years. Tumour cells showed cytoplasmic expression of vimentin and were negative to desmin, α-SMA, S-100 and c-Kit. Leiomyosarcomas were diagnosed in one female (14y.) and one male (16 y.). Tumour cells showed cytoplasmic expression of vimentin and α-SMA and were negative to desmin, S-100 and c-Kit. GISTs were confirmed in one female (9y.) and one male (10y.) Tumour cells expressed DOG1, c-Kit, α-SMA and vimentin, and were negative to desmin and S-100. Non-GIST, non-smooth muscle NIMT was diagnosed in a 6-year-old male dog. Tumour cells expressed vimentin, S-100 and NSE and were negative to CD3, CD20, CD79a, MHC II, MUM1, desmin, c-Kit, pancytokeratin and α-SMA. In the group of sarcomas, no association with breed was identified. Lymphomas included 6 cases of B-cell lymphoma (5 centroblastic and one centroblastic polymorphic, Fig. 3) and 3 cases of T-cell lymphoma (2 cases of large T-cell lymphoma and one case of lymphoblastic T-cell lymphoma). Lymphomas were diagnosed in 7 males and 2 females, with an age ranged from 3 to 12 years (mean age: 6.9y.; SD ± 2.9). In B-cell lymphomas, tumour cells showed cytoplasmic expression of CD20 and CD79a and were negative to CD3. All of them were diagnosed in males with a mean age of 6.2 years. In T-cell lymphomas, tumour cells showed cytoplasmic expression of CD3 and were negative to CD20 and CD79a. This group was represented by 2 females and one male with a mean age of 9 years. In lymphomas, no breed predisposition was observed. Plasmacytomas were diagnosed in two males and one female, with an age ranged from 11 to 12 years (mean age: 11.7y.; SD ± 0.6).

**Fig. 4:**
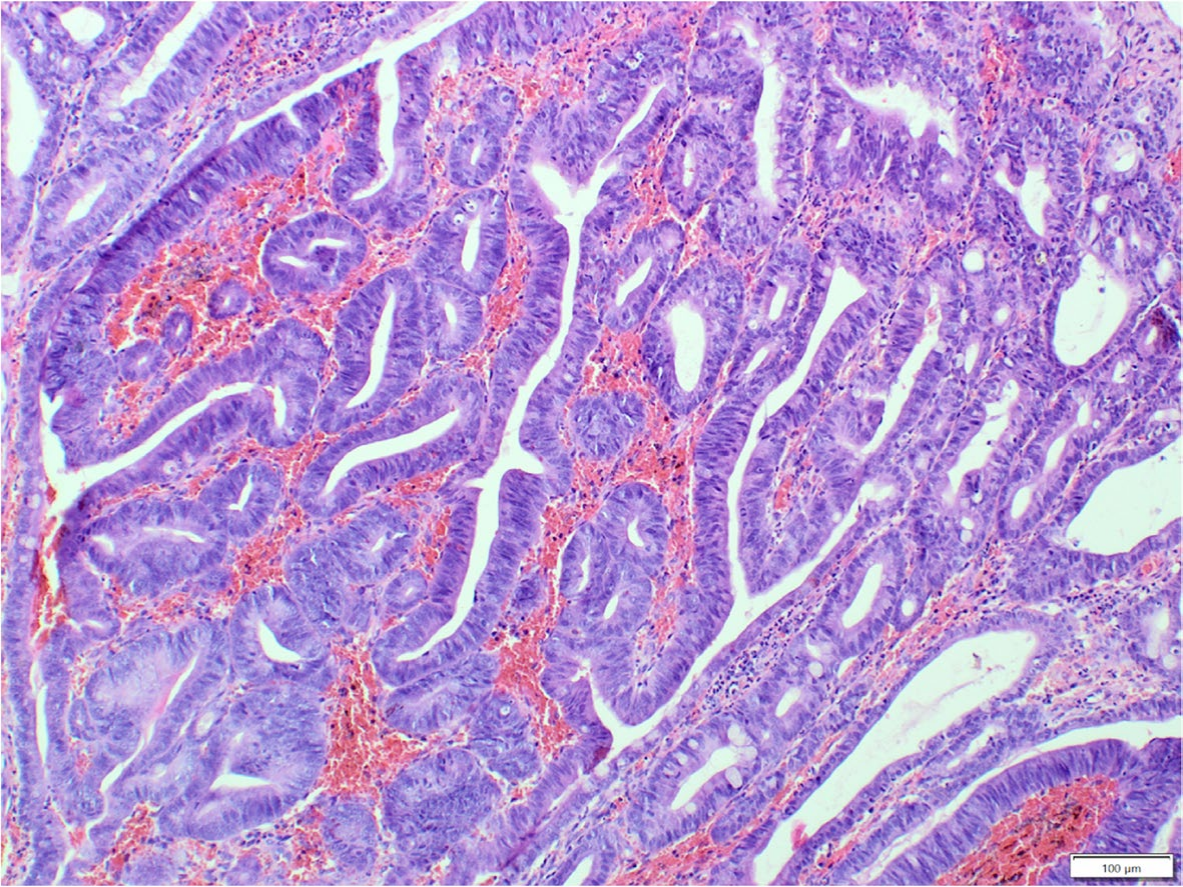
Dog, adenoma, large intestine. The tumour cells are oval with moderate anisocytosis and anisokaryosis, form irregular crypts, sometimes widened. Some of the glands are with double or multilayer formation. The stroma with strong congestion, infiltrated by lymphocytes, plasma cells and neutrophils. Hematoxylin and eosin (HE)

Benign tumours constituted 26.7% (58/217) of the total cases. The most frequently diagnosed benign tumour was adenoma (52/217, Fig. 4), observed more often in males (32/52) than in females (20/52). The age of the affected animals ranged from 1 to 15 years (mean age: 5.9y.; SD ± 3.8), and the most frequently affected breed was the French Bulldog (15/37), followed by crossbreeds (5/37) and West Highland White Terriers (4/37). Unfortunately, these data were incomplete in several cases: 5 regarding the age and 15 regarding the breed. Benign mesenchymal tumours were less numerous than adenomas and were represented by leiomyomas (6/217), observed in five females and one male, with an age ranged from 7 to 13 years a mean age of 10 years (SD ± 2.2).

**Fig.5:**
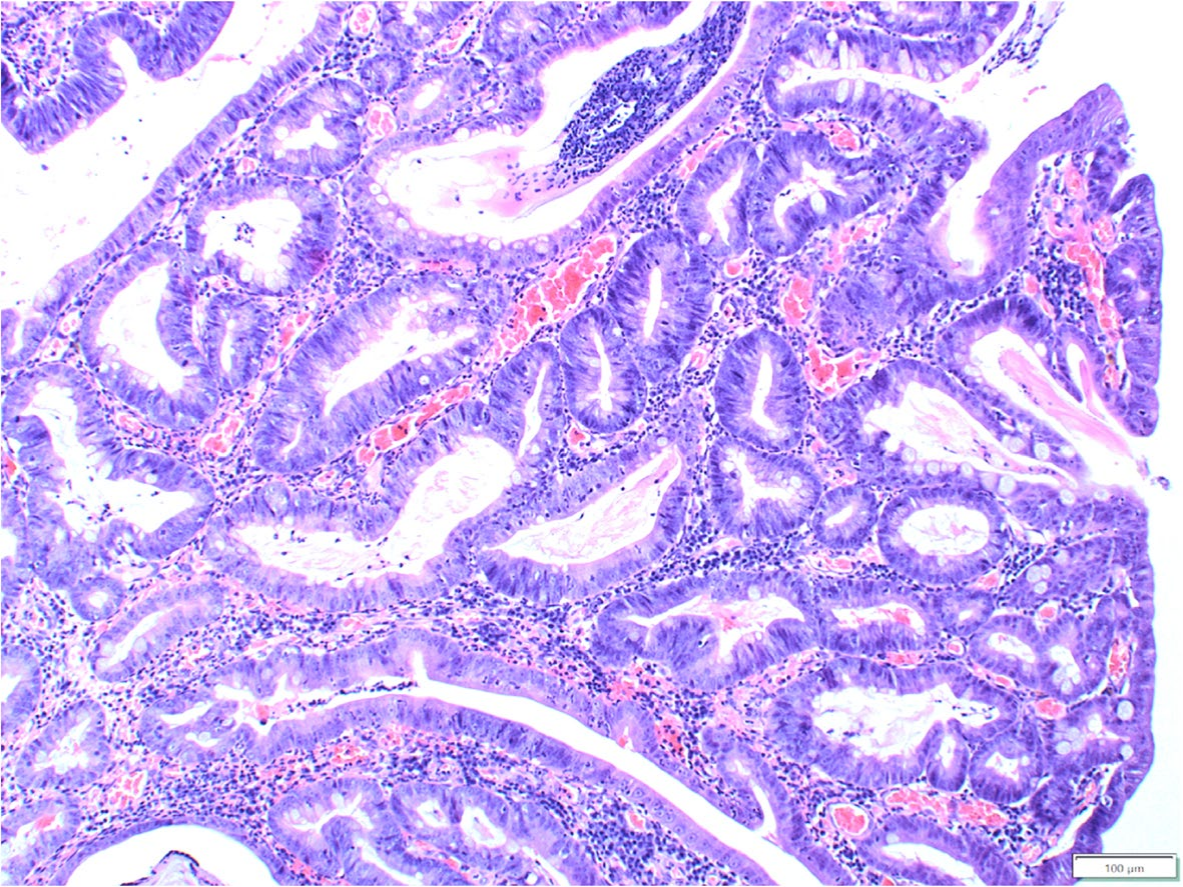
Dog, hyperplastic polyp, large intestine. Uniform columnar layer of cells with minor anisocytosis and anisokaryosis, form irregular crypts, occasionally filled with mucinous secretion. The stroma is composed of collagen-rich connective tissue, with congestion, occasionally heavily infiltrated with plasma cells, lymphocytes, variably numerous neutrophils, and eosinophils. Hematoxylin and eosin (HE)

**Fig. 6:**
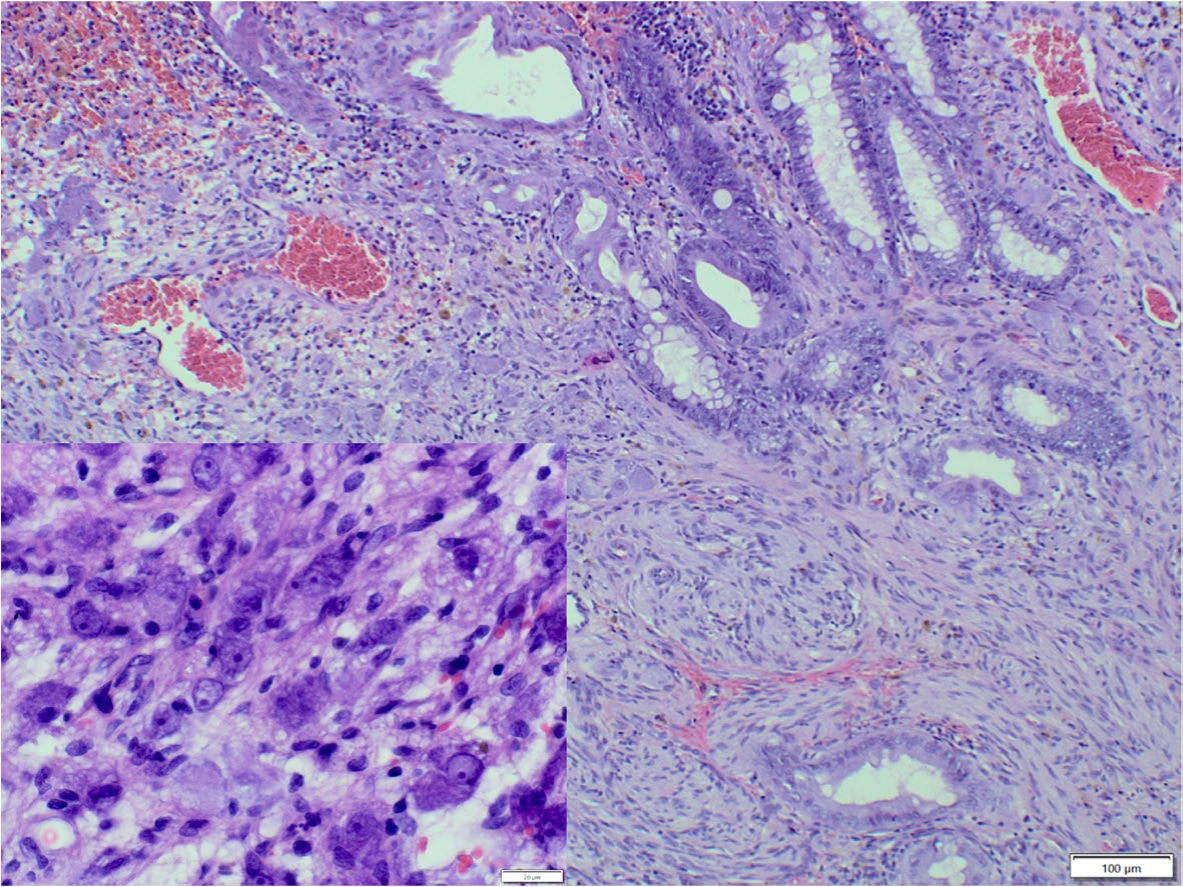
Dog, Ganglioneuromatosis in hamartomatous polyp, large intestine. Multifocal proliferation of ganglion cells, (inset) accompanied by proliferation of fibrous connective tissue. Dilated, congested blood vessels. Hematoxylin and eosin (HE)

Non-neoplastic colorectal proliferative lesions constituted 21.1% (45/217) of the total cases. In this heterogeneous group of colorectal polyps, hyperplastic polyps were the most numerous (21/217, Fig. 5), followed by inflammatory polyps (12/217), fibroblastic polyps (5/217), lymphoid polyps (4/217) and two hamartomatous polyps. Hyperplastic polyps were observed more often in males (13) than in females (8), with an age ranged from 2 to 14 years mean age of 6.3 years (SD ± 3.8), and more often in French Bulldogs (5 cases), Yorkshire Terriers (2 cases) and crossbreeds (2 cases). Inflammatory polyps were observed more often in females (9) than in males (3), with an age ranged from 2.8 to 10 years and mean age of 8 years (± 3.1). No breed predisposition was identified. Fibroblastic polyps were diagnosed only in females, with an age ranged from 1 to 13 years and mean age of 7.8 years (SD ± 3.8), without any breed predisposition. Sex distribution was equal in lymphoid polyps, with an age ranging from 3 to 14 years and mean age of 9.3 years (SD ± 5.6), no breed predisposition was observed. One case of hamartomatous polyp was diagnosed in a 7-year-old female French Bulldog and the second one in a 4-year-old male Jack Russell Terrier; the latter was accompanied by ganglioneuromatosis (Fig. 6).

The statistical analysis did not confirm significant differences between occurrence of non-neoplastic vs. neoplastic lesions depending on sex, breed, or age (*p* > 0.05 for all variables). However, dogs with benign tumours were younger (6; SD ± 3.8 years) than dogs with malignant tumours (8.25; SD ± 3.30 years; *p* < 0.001), and this observation was confirmed as well between adenomas (5.9y.; SD ± 3.8) and adenocarcinomas (8y.; SD ± 3.22; *p* < 0.001).

Additionally, benign tumours were more frequently diagnosed than malignant tumours in French Bulldogs (*p* = 0.017). In the group of adenomas (37 cases with breed data available), a comparison between the common breeds represented in this cases series demonstrated that French Bulldogs were predominant (*p* = 0.025). Logistic regression analysis confirmed these findings, showing that age and French Bulldogs breed were significant predictors for occurrence of malignant tumours vs. benign tumours. The odds of malignant tumours increased by 21% with each year of age (OR = 1.21, CI95 [1.09–1.35], *p* < 0.001), except in the French Bulldogs in which the odds of malignant tumour is reduced by 75% compared to other breeds (OR = 0.25, CI95 [0.10–0.60], *p* = 0.002).

The breeds most frequently affected by selected tumours and tumour-like lesions were presented in Table 2. Detailed information, including number of cases, sex distribution and mean age were collected in Table 3.

**Table 2:** The most common dog breeds observed in canine colorectal proliferative lesions. Lesions were selected based on the number of cases (at least 15) with breed information

Proliferative lesion	Number of total cases with breed information	Number of cases (%)	Breed
Adenocarcinoma	67	10 (14.9%)	French Bulldogs
		7 (10.4%)	Yorkshire Terriers
		7 (10.4%)	Crossbreeds
Adenoma	37	15 (40.5%)	French Bulldogs
		5 (13.5%)	Crossbreeds
		4 (10.8%)	West Highland White Terriers
Hyperplastic polyp	19	5 (26.3%)	French Bulldogs
		2 (10.5%)	Yorkshire Terriers
		2 (10.5%)	Crossbreeds

**Table 3:** Frequency, sex distribution and mean age of individual lesions

Diagnosis	Number of cases (%)	Female	Male	Mean age (years)
**Neoplastic:** malignant	114 (52.5%)	44	70	8.3
Adenocarcinoma	93 (42.9%)	38	55	8
Lymphoma	9 (4.1%)	2	7	6.9
B-cell type	6 (2.8%)	0	6	6.2
T-cell type	3 (1.4%)	2	1	9
Sarcoma	9 (4.1%)	3	6	10.9
Fibrosarcoma	4 (1.8%)	1	3	10.7
Leiomyosarcoma	2 (0.9%)	1	1	15
Gastrointestinal stromal tumour (GIST)	2 (0.9%)	1	1	9.5
Non-GIST, non-smooth muscle (NIMTs)	1 (0.5%)	0	1	6
Plasmacytoma	3 (1.4%)	1	2	11.7
**Neoplastic:** benign	58 (26.7%)	25	33	6
Adenoma	52 (24.0%)	20	32	5.9
Leiomyoma	6 (2.8%)	5	1	10
**Non – neoplastic lesion**	45 (21.1%)	25	20	7
Heterogeneous colorectal polyps	44 (20.3%)	25	19	7.1
Hyperplastic polyps	21 (9.7%)	8	13	6.3
Inflammatory polyps	12 (5.5%)	9	3	8
Fibroblastic polyps	5 (2.3%)	5	0	7.8
Lymphoid polyps	4 (1.8%)	2	2	9.3
Hamartomatous polyps	2 (0.9%)	1	1	5.5
Ganglioneuromatosis	1 (0.5%)	0	1	4

## Discussion

Neoplastic and non-neoplastic lesions of the gastrointestinal tract, especially of the large intestine, are not commonly diagnosed in dogs. Meanwhile, in humans, due to the widespread use of preventative screening tests, such as colonoscopy, these diseases can be diagnosed at an early stage and successfully treated. Hopefully, the development and improvement of diagnostic procedures in veterinary medicine, together with the increase in the longevity of dogs and the awareness of owners, will contribute to the rise in detection of colorectal lesions in dogs as well. Hence, it is very important to conduct retrospective studies assessing the frequency of occurrence and precise characteristics of colorectal proliferative neoplastic and non-neoplastic lesions in dogs, especially since the number of such studies in the available literature is limited.

In this retrospective study, conducted on a large number of histologic samples, benign tumours were diagnosed in younger dogs, compared to malignant tumours. This finding was also confirmed in epithelial tumours, between adenomas and adenocarcinomas. To the best of our knowledge, we showed for the first time the relationship between the age of the animals and malignancy of the colorectal tumours. However, this finding is not surprising, as possible progression of colorectal adenomas to adenocarcinomas over time has been previously reported [15, 59, 62].

In veterinary literature, a higher frequency of tumours in males in the canine large intestine has already been observed [21, 41, 48]. Our findings confirm these statements, as we noted overrepresentation of males in malignant tumours, as well as in adenomas. However, among all the lesions in the large intestine, there were no statistical differences regarding sex predilection.

We have demonstrated, in our study, that half of the proliferative colorectal lesions in dogs were diagnosed as malignant tumours, mainly of epithelial origin. Not surprisingly, adenocarcinoma was the most frequently diagnosed lesion, which is in line with the previous study [41]. We observed that colorectal adenocarcinomas were mostly of the papillary type, in contrast to results indicating that the tubular type was the most common [3, 49]. Furthermore, we also diagnosed single cases of uncommon histological types, such as mucinous adenocarcinoma and adenosquamous carcinoma. We observed that males were overrepresented in colorectal adenocarcinoma, which is consistent with most previous reports [33], [55], [52], although in one study females were more frequently affected [16]. The mean age of animals affected by adenocarcinoma was 8 years,a similar finding was observed previously [16, 52]. Recent studies revealed that German Shepherds, Collies, Jack Russell Terriers, and Miniature Dachshunds are potentially predisposed to colorectal adenoma [37, 49], nevertheless we did not observe a similar breed predisposition in our study.

In the present study, the second most common colorectal lesion was benign epithelial tumour—adenoma. In dogs, the large intestine (and more precisely – distal rectum) is the most common site for the development of this type of lesion [55]. We observed a slightly higher incidence of large intestine adenoma in males, which is consistent with another survey [55]. In previous reports the mean age of affected animals was 7–8 years [46, 49]. However, in our study adenomas occurred in younger dogs, with the mean age 5.9 years, and 9 cases were diagnosed in very young dogs (1–2 years old). Interestingly, we observed for the first time that French Bulldogs were a predominated breed among colorectal adenomas. Additionally, the incidence of colorectal adenoma in this breed was significantly higher, compared to adenocarcinoma. Although a previous study suggested that Miniature Dachshunds are prone to developing colorectal adenomas [49] however our results did not support this finding.

Colorectal polyps were the third most common lesion found in the present study with a predominance of hyperplastic polyps, followed by inflammatory polyps, fibroblastic polyps, lymphoid polyps and hamartomatous polyps. Fibroblastic polyp described in humans has been recently reclassified as perineurioma, due to characteristic immunohistochemical features [58] and has no equivalent in veterinary medicine. Colorectal polyps were observed more commonly in females, in contrary to the previous studies, which stated than colorectal polyps were more common in males [32, 38] or sex distribution was almost equal [50, 54]. However, in the present study, the sex distribution varied depending on the type of polyp,thus, hyperplastic polyps were more common in males, inflammatory polyps – in females, while fibroblastic polyps occurred exclusively in females. In lymphoid and hamartomatous polyps sex distribution was equal. In the present study, the mean age of dogs affected by non-neoplastic colorectal polyps was 7 years, which is in line with the previous studies [32, 38]. However, we observed that hyperplastic polyps occurred in younger animals (mean age 6.3 years) than other types of polyps (mean age range 7.8–9.3 years). Furthermore, in contrast to previous studies, which identified Miniature Dachshunds, Jack Russel Terriers and West Highland White Terriers as predisposed breed to the development of colorectal polyps [32, 38, 61] we did not any detect any breed predispositions to these lesions in our study.

Surprisingly, both hamartomatous polyps, described in the present study, occurred in mature dogs (4 and 7 years-old), while these polyps were previously found only in puppies [4, 14]. In humans, hamartomatous polyps are usually diagnosed in children or young adolescents and they can occur spontaneously or be inherited as part of Peutz-Jeghers syndrome and juvenile polyposis syndrome [2]. One of the colorectal hamartomatous polyps, associated with ganglioneuromatosis, was diagnosed in a Jack Russell Terrier, which may correspond with the previous research indicating hereditary gastrointestinal polyposis in this breed [60, 61]. The second case of colorectal hamartomatous polyp in our study was diagnosed in the French Bulldog—the breed which was the most frequently identified among all colorectal lesions.

Non-epithelial malignant colorectal tumours in the present study were represented by lymphomas and sarcomas. Although T-cell lymphomas predominate among gastrointestinal tract lymphomas in dogs (Ozaki et al. 2006; [45]), B-cell lymphomas occur more often in the large intestine [12, 56], what was also observed in the present study. Moreover, diffuse large B-cell lymphoma is the most common type of lymphoma in colon also in humans [44]. We observed a higher incidence of colorectal lymphoma in males, as opposed to reports where the sex distribution was equal in both genders [12, 56]. The mean age of dogs with colorectal lymphomas was 6.9 years, which is consistent with one of the previous studies [56] and is slightly higher than in another study [12]. We did not find any breed predilection to colorectal lymphoma, which is consistent with previous observation [12].

The results of our study revealed that sarcomas were far less common in the large intestine, compared to adenocarcinomas, and were represented by fibrosarcomas predominating, followed by two cases of leiomyosarcomas and GISTs. Occurrence of mesenchymal tumours in the canine gastrointestinal tract was the subject of extensive study [5, 17, 31], especially their immunohistochemical reclassification between GISTs and leiomyosarcomas [10, 20], [11]. In the literature, there is limited information regarding the observation of fibrosarcoma in the canine large intestine. Interestingly, we diagnosed this type of tumour more frequently than leiomyosarcomas and GISTs. It was previously stated that GISTs developed more often in the large intestine than in other locations of the canine gastrointestinal tract [17, 47], however in our study, only two cases were classified as GIST. Whereas, the previous statement, that canine leiomyosarcomas occurred more frequently in the stomach and small intestine [47] is consistent with the fact that we diagnosed only two cases of this lesion in the canine large intestine. We observed that sarcomas were diagnosed in older dogs, with the mean age of approximately 11 years. Previous studies revealed the similar mean age of dogs with intestinal leiomyosarcomas [8, 27]. However, both leiomyosarcomas included in the present study were diagnosed in dogs even older. Furthermore, the mean age of the affected dogs with GIST in our study was 9.5 years, which is slightly lower compared to the previous results which were around 11 years [17, 18]. All subtypes of sarcomas were observed in females and males almost equally without any notable breed predilection. Another tumour, which was diagnosed in the canine large intestine was extramedullary plasmacytoma. Based on the current veterinary literature, plasmacytoma occasionally developed in the colorectal area of the older animals, with the mean age ranging from 9.4 to 9.7 years [29], though we observed an even higher mean age of 11.7 years. No gender or breed predilection for the occurrence of colorectal plasmacytoma was observed in our study.

Although the most common location for leiomyoma of the gastrointestinal tract in dogs is the stomach [17, 24], we observed 6 leiomyomas in the large intestine, classifying them as the second most common benign tumour, after adenomas. In the present study, leiomyoma was diagnosed with the mean age of 10 years, which is consistent with the previous report [17]. However, it was previously demonstrated that leiomyomas of the gastrointestinal tract occur more commonly in males [17], while we observed higher incidence of this benign mesenchymal tumour in females. Among cases diagnosed with leiomyoma, no breed predisposition was observed.

Additionally, our study confirms that in the canine large intestine, the rectum is the typical location for proliferative lesions, both non-neoplastic and neoplastic, which is consistent with previous studies [1, 7, 49]. Presumably, the rectum is easier to assess during direct clinical examination compared to colon, in response to clinical signs such as hematochezia, dyschezia and tenesmus through rectal palpation. Even could be incidentally discovered during anal sacs emptying. To diagnose lesions in the colon, additional diagnostic techniques such as ultrasound or computed tomography are needed and when mass is present, colonoscopy of the entire large intestine is recommended, which can be problematic for some owners due to additional costs [1, 42]. However, recent veterinary studies have presented the positive effects of application percutaneous ultrasound-guided fine-needle aspirates to differentiate types of lesions in the gastrointestinal tract, including the colon, which can be a less expensive alternative in certain cases [22].

## Conclusion

In summary, based on the conducted study, we have updated current knowledge regarding colorectal proliferative lesions in dogs. The vast majority of colorectal tumours in dogs were of epithelial origin, with the most common adenocarcinomas, followed by adenomas. Both adenocarcinomas and adenomas were more commonly observed in males (and in general category of malignant tumours), and adenoma was diagnosed in younger dogs, compared to adenocarcinoma. Thise correlation was also observed in the term of benign tumours versus malignant tumours.

Colorectal polyps were the third most common group of lesions, with a higher incidence of hyperplastic polyps, which were more frequently diagnosed in males, followed by inflammatory and fibroblastic polyps, often observed in females. Additionally, we observed for the first time an overrepresentation of French Bulldogs in colorectal lesions, especially in adenomas, and these results can be a basis for further studies to investigate genetic factors in this specific breed. Non-epithelial tumours occurred infrequently in the large intestine in dogs, but leiomyomas were the second most commonly observed benign tumour in this localization. Lymphomas occurred quite infrequently but were mostly of B-cell type, often diagnosed in males. Moreover, the relationship between age and malignancy probably reflects the possible malignant transformation of benign tumours with age in dogs. Therefore, the results of our research are alarming and emphasize the importance of routine diagnostics of each colorectal lesion in canine large intestine.

## Data Availability

Data used in the current research are available from the corresponding author on reasonable request.
